# Identifying Predictors of Utilization of Skilled Birth Attendance in Uganda Through Interpretable Machine Learning

**DOI:** 10.3390/ijerph22111691

**Published:** 2025-11-09

**Authors:** Shaheen M. Z. Memon, Robert Wamala, Ignace H. Kabano

**Affiliations:** 1 African Centre of Excellence in Data Science, College of Business and Economics, University of Rwanda, Kigali P.O. Box 4285, Rwanda; i.kabano@ur.ac.rw; 2 Department of Planning and Applied Statistics, College of Business and Management Sciences, Makerere University, Kampala P.O. Box 7062, Uganda; wamalar@gmail.com

**Keywords:** maternal healthcare, skilled birth attendance, machine learning, class imbalance, class weights, SHAP explainability

## Abstract

Skilled Birth Attendance (SBA) is essential for reducing maternal and neonatal mortality, yet access remains limited in many low- and middle-income countries. This study used machine learning to predict SBA use among Ugandan women and identify key influencing factors. We analyzed data from the 2016 Uganda Demographic and Health Survey, focusing on women aged 15 to 49 who had given birth in the preceding five years. After preparing and selecting relevant features, six tree-based models (decision tree, random forest, gradient boosting, XGBoost, LightGBM, CatBoost) and logistic regression were applied. Class imbalance was addressed using cost-sensitive learning, and hyperparameters were tuned via Bayesian optimization. XGBoost performed best (F1-score: 0.52; recall: 0.73; AUC: 0.75). SHapley Additive Explanations (SHAP) were used to interpret model predictions. Key predictors of SBA use included education level, antenatal care visits, region (especially Northern Uganda), perceived distance to a healthcare facility, and urban or rural residence. The results demonstrate the value of interpretable machine learning for identifying at-risk populations and guiding targeted maternal health interventions in Uganda.

## 1. Introduction

Maternal health services including antenatal care (ANC), Skilled Birth Attendance (SBA), and postnatal care are critical interventions that reduce maternal and neonatal mortality [1,2,3]. SBA, as defined by the World Health Organization (WHO), requires the presence of a trained and accredited health professional, such as a midwife, doctor, or nurse, during labor, delivery, and the immediate postpartum period [4]. These trained professionals play a key role in preventing and managing obstetric emergencies like obstetric haemorrhage, hypertensive disorders in pregnancy, and pregnancy-related infections, which are the leading causes of maternal death in Sub-Saharan Africa [5]. Their presence at birth also ensures good hygiene, timely referral in the event of complications, and continuous care, ultimately protecting mothers and infants from morbidity and mortality [6]. Widespread utilization of SBA greatly reduces deaths and complications of both mother and newborn, making better access to skilled birth attendants essential for improving maternal and child health worldwide [4].

In spite of the clear need of SBA during delivery, disparities in healthcare access, infrastructure, wealth, and education contribute to inconsistencies in utilization in many low- and middle-income countries [7,8,9]. Despite efforts to promote facility-based deliveries, many births in Sub-Saharan Africa still occur without the presence of a qualified health professional, increasing the risks of complications and adverse maternal and neonatal outcomes [10].

Uganda implemented a skilled attendance at birth strategy as part of its efforts to meet the Millennium Development Goal (MDG) 5 target of reducing the maternal mortality ratio (MMR) by 75% [11]. This initiative led to a significant increase in skilled birth attendance, rising from 35% in the 1990s to 42% in 2006, and reaching 74% by 2016. However, this progress still fell short of the 90% coverage target [12]. Similarly, Uganda’s Maternal Mortality Rate (MMR) declined from 506 maternal deaths per 100,000 live births in 1995 to 336 deaths per 100,000 live births in 2016 [13], yet remained far from the stated goal of reducing the MMR by 75%. The challenges in reaching this goal highlight the need for more targeted, data-driven approaches to identify women at risk of not using skilled delivery services and develop interventions that increase uptake [14,15].

Traditional statistical methods, such as logistic regression, have been widely used to study the determinants of SBA use, but these methods rely on assumptions like linearity and distributional form that may not hold in complex datasets. These methods are mainly suited for inference, focusing on estimating associations and testing hypotheses. Furthermore, studies that have employed methods such as logistic regression usually do not test for predictive performance. Machine Learning (ML), on the other hand, is designed for prediction, using flexible algorithms that can model non-linear relationships and high-dimensional data without strict parametric assumptions [14,15].

This study aimed to use interpretable machine learning methods to predict Skilled Birth Attendance (SBA) among women of reproductive age in Uganda and to identify the key socio-demographic, economic, and obstetric factors influencing its use. The primary outcome variable was SBA use, coded as a binary measure (Yes = delivery assisted by a skilled health provider; No = delivery without a skilled attendant). Using nationally representative data from the 2016 Uganda Demographic and Health Survey (UDHS), we applied and compared multiple models, including logistic regression, decision tree, random forest, gradient boosting, XGBoost, LightGBM, and CatBoost, to identify the model with the best performance in the prediction of SBA use. Predictions from the best-performing model were then analyzed using SHapley Additive Explanations (SHAP) to determine the most influential predictors of SBA use and to examine whether each factor increased or decreased the likelihood of using skilled delivery services. By combining prediction and interpretability, the study provides policy-relevant insights that can inform targeted maternal health interventions in Uganda.

## 2. Related Literature

Previous studies from across sub-Saharan Africa have identified a range of socio-demographic, economic, and obstetric factors associated with the use of skilled birth attendance (SBA) or health facility delivery. Maternal education is consistently reported as one of the most influential determinants, with women who have attained secondary education or higher being significantly more likely to seek skilled care during childbirth [16,17]. Antenatal care (ANC) attendance, especially completing at least four visits, has also been strongly linked to increased SBA use, as it promotes early identification of complications and strengthens engagement with the healthcare system [18,19]. Geographic location plays a critical role; studies from Sub-Saharan Africa show that urban women consistently report higher utilization of skilled delivery services than their rural counterparts, largely due to better access to healthcare infrastructure [10,20]. Wealth status, often captured through the household wealth index, is another key predictor, with wealthier women more likely to afford transportation and delivery-related costs [21]. Furthermore, perceived distance to health facilities and partner’s educational attainment have also been found to influence SBA uptake, particularly in African contexts, where male partners often play a role in healthcare decision-making [22,23].

Studies on SBA have predominantly used traditional statistical methods [8,9,24,25,26,27], which may not fully capture the complex relationships and interactions among socio-economic, demographic, and obstetric factors that influence the uptake of SBA. Machine Learning (ML) models have demonstrated success in identifying patterns in maternal health data, providing valuable insights into which women are most at risk of not using maternal health services [28,29,30,31].

A few studies on SBA use and place of delivery have been done using ML methods on multi-country data. Ngusie et al. [32] used data from 12 Sub-Saharan countries in the prediction of place of delivery and Taye et al. [33] used data from 27 African countries in the prediction of SBA use. On the other hand, Fredriksson et al. [29] used data from only one city in Tanzania in the prediction of place of delivery. This study focused solely on prediction and did not utilize explainability tools to determine the top influencers of women’s decisions. In certain studies, place of delivery was used as a predictor for SBA use [30,33]. Because this variable is inherently related to the outcome, models that include it may appear to perform better than those relying only on predictors available before delivery.

While some studies have applied ML to the prediction of place of delivery and SBA use, our study makes several contributions: it focuses on nationally representative data from Uganda to provide country-specific insights, applies tree-based models with class weighting to address imbalanced categorical data, and goes beyond prediction by using explainability tools such as SHAP with visualization techniques to interpret the key drivers of SBA use.

## 3. Methodology

### 3.1. Data Source

This study used secondary data from the 2016 Uganda Demographic and Health Survey (UDHS), a nationally representative cross-sectional survey conducted by the Uganda Bureau of Statistics (UBOS) in collaboration with ICF under the DHS Program. Data collection took place between June and December 2016 using standardized DHS protocols. The survey used a two-stage stratified sampling design, with enumeration areas selected from the 2014 National Population and Housing Census as the sampling frame, followed by systematic household selection. The survey covered 19,588 households across 15 regions of Uganda, capturing both urban and rural areas. A total of 18,506 women aged 15–49 and 5336 men aged 15–54 were successfully surveyed using structured questionnaires programmed on tablets to ensure quality and completeness [13]. Information collected included household characteristics, fertility, family planning, maternal and child health, and health service utilization.

### 3.2. Study Population

**Figure 1:**
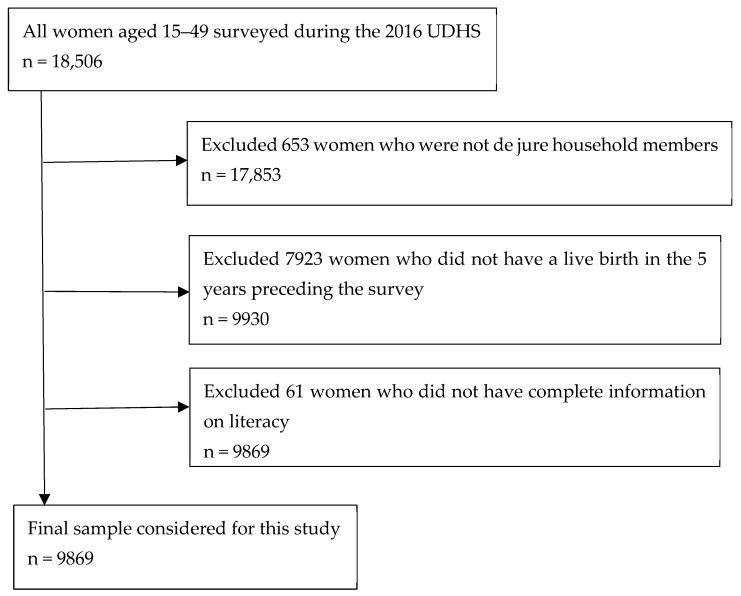
Flowchart of sample selection from the 2016 Uganda Demographic and Health Survey.

Figure 1 shows the process of selecting women eligible for inclusion in this study from the 2016 UDHS dataset based on the following criteria:

Inclusion criteria: Women aged 15–49 years who were de jure household members (because they have complete and reliable information on key household variables used in the analysis), had at least one live birth within five years preceding the survey (to minimize recall bias), and had complete information on literacy were included in this study.

Exclusion criteria: Women who were visitors in the household, those without a live birth during the reference period, and those without information on literacy were excluded from the study.

The resulting subset constituted the analytic dataset for this study.

### 3.3. Study Variables

The variables used in this analysis were derived from the UDHS Woman’s Questionnaire, which includes standardized questions on maternal health service utilization, socio-demographic background, household characteristics, and obstetric attributes. The selection and organization of explanatory variables (features) from the original 2016 UDHS data were guided by Andersen’s Healthcare Utilization Model, which groups determinants of healthcare-seeking behaviors into three domains [34]. This framework was applied to explain how individual, household, and contextual characteristics influence a woman’s decision and ability to seek skilled delivery services. In this study, predisposing, enabling, and need factors were used to model and predict the likelihood of utilizing SBA.

(i) Predisposing factors: These capture socio-demographic and cultural characteristics that influence an individual’s likelihood to use health services. Variables included: sex of household head, age of household head, marital status, family type, household size, region, family mobility, religion, education level, literacy, frequency of reading newspaper, frequency of listening to radio, frequency of watching tv, partner’s education level, fertility preference, age first sex, age first birth, children ever born, age group, and birth interval.
(ii) Enabling factors: These are the economic or logistic resources or conditions that facilitate or hinder access to healthcare. Variables included: owning a bank account, wealth index, internet use, health insurance, radio ownership, television ownership, mobile ownership, place of residence, perceived distance to healthcare facility, perceived healthcare cost, partner’s employment status, healthcare autonomy, expenditures autonomy, and employment status.
(iii) Need factors: These are the individual’s perception of their health status and their perceived need for healthcare. Variables included: wanted pregnancy, pregnancy duration, number of ANC visits, first trimester ANC, contraception use, and prior healthcare facility visits.

In addition, the Sex Marriage Birth (SMB) sequence variable was also examined as a predictor of Skilled Birth Attendant (SBA) use. This variable represents the order in which key life events, i.e., sex, marriage, and childbirth, occur in a woman’s life. It may provide additional contextual and behavioral insights, offering a broader perspective than individual factors like age at first sex or age at first birth. The SMB sequence can also reflect social norms that influence maternal healthcare decisions.

The variable includes the following categories:

(i) Sex → Marriage → Birth (SMB);
(ii) Marriage → Sex → Birth (MSB);
(iii) Sex → Birth → Marriage (SBM);
(iv) Sex → Birth → No Marriage (SBNoM).

The main outcome variable (target) in this study was: use of a Skilled Birth Attendant (SBA) during the most recent delivery, coded as a binary variable; Yes—delivery assisted by a skilled health provider (doctor, nurse, midwife, medical assistant, or clinical officer), and No—delivery assisted by an unskilled person or no attendant.

**Figure 2:**
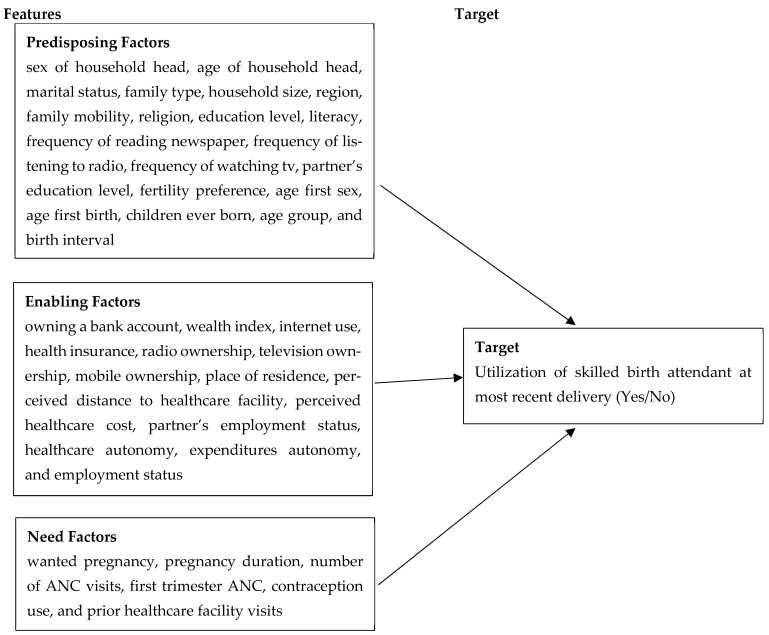
Conceptual framework based on Andersen’s Healthcare Utilization Model for predicting utilization of SBA in Uganda.

Figure 2 presents the conceptual framework guiding the selection and organization of variables used to predict SBA utilization.

### 3.4. Data Preprocessing

First, a few outliers in the variables were assessed using the Interquartile Range (IQR) method and were dropped. Missing data were imputed using a K-Nearest Neighbors (KNN) imputation approach, which estimates missing values based on the similarity of observations within the dataset. For each categorical feature with missing values, KNN identifies the k most similar observations (nearest neighbors) using an appropriate distance metric for categorical data, such as Hamming distance. The missing value is imputed based on the most frequently occurring category among the k-nearest neighbors. This process is repeated for all variables with missing values. KNN imputation has been shown to achieve higher predictive accuracy for missing data compared to other imputation methods [35,36].

Next, features were assessed for being near constant. A near-zero variance variable is one in which nearly all observations fall into a single category, leaving very little variability. This extreme imbalance means that almost every observation has the same value (e.g., “no”), which offers little information for differentiating between observations. Consequently, this variable is considered to have near-zero variance and may not be useful for predictive modeling [37]. Multicollinearity among categorical variables was assessed using Cramer’s V, which measures the association between two nominal variables on a scale from 0 (no association) to 1 (perfect association). Finally, two types of encodings were applied depending on the nature of the categorical features; one-hot encoding was used on nominal variables, creating a separate dummy variable for each unique category. Label encoding was used on ordinal variables to map each category to an integer in a way that maintains the ordering. This step transforms categorical data into numeric form, which is compatible with most machine learning algorithms.

### 3.5. Data Splitting and Handling Class Imbalance

To enable reliable model evaluation, the dataset was split into a training set (80%) for model training, and a testing set (20%) for evaluating model performance on unseen data. The split was performed randomly via stratified sampling while preserving the distribution of the target variable.

Class imbalance can skew machine learning models, favoring the majority class and leading to poor predictive performance for the minority class. To deal with this issue, a cost-sensitive learning approach was applied by assigning higher misclassification penalties to the minority class. This approach ensured that the learning algorithm prioritized the underrepresented class without artificially altering the dataset’s distribution [38].

In our approach, we used class weights during model training computed using the formula:

$$Weight\;for\;Class\;0\;\left(minority\right)=\frac{Total\;Samples}{2\times Number\;of\;samples\;in\;class\;0}\;Weight\;for\;Class\;1\;\left(majority\right)=\frac{Total\;Samples}{2\times Number\;of\;samples\;inclass\;1}$$

This formula assigns higher weights to the minority class by accounting for the imbalance in the dataset. By applying these weights, we ensured that the model focused more on the minority class during training, helping to balance the influence of both classes. This method improves the model’s ability to predict the minority class more effectively without being dominated by the majority class. The cost-sensitive weighting was consistently applied across both feature selection and model training. Because our dataset consisted entirely of categorical predictors, we relied on class weighting rather than resampling methods such as SMOTE or ADASYN. These methods generate synthetic values by interpolating between existing observations, which works well with continuous data but produces unrealistic cases when applied to categorical variables (e.g., “half urban, half rural”) [39,40]. For this reason, class weighting was the more appropriate strategy for our study.

### 3.6. Feature Selection

We used Elastic Net for feature selection because it effectively manages correlated predictors while retaining the most important ones. The method blends L1 (Lasso) and L2 (Ridge) regularization to shrink less useful coefficients toward zero, reducing overlap among features. Unlike Lasso, which can randomly remove one variable from a correlated group, Elastic Net shares weight among related predictors and drops only those that contribute minimal new information [41]. Although tree-based models like Random Forest and XGBoost can tolerate multicollinearity, using Elastic Net before model training reduces redundancy, improves computational efficiency in the final modelling, and makes the selected features easier to interpret.

### 3.7. Model Training

This study focuses on the use of machine learning models that provide not only strong predictive capability but also clear interpretability. Our goal is not just to predict outcomes but to understand the drivers behind them, and to do so in a way that can be clearly communicated to policymakers, practitioners, and stakeholders. For that reason, we focused on models that avoid the black-box nature of many machine learning techniques and instead offer transparent, explainable decision-making.

We used a variety of tree-based algorithms for their well-established strength in capturing complex patterns while still providing clear insights into which factors drive predictions. These models enable us to identify the most important predictors of skilled birth attendance, explain individual-level predictions, and translate findings into actionable policy recommendations. These methods have been briefly outlined below:

Decision Trees: Non-parametric supervised learning models used for classification and regression. They split data into smaller groups based on simple, interpretable rules derived from feature values, forming branches that lead to terminal nodes representing predictions. They are easy to interpret and visualize, and they handle both numerical and categorical data with minimal preprocessing. However, deep or highly complex trees can overfit, so pruning or limiting tree depth is often used to improve generalization. Although single trees can be sensitive to small variations in data, combining multiple trees in ensemble methods such as Random Forest or Gradient Boosting improves both accuracy and stability [42].

Random Forest: An ensemble approach that builds many decision trees and combines their outputs to produce more accurate and stable results in both classification and regression. Each tree is trained on a random sample of the data and a random selection of features, which introduces variation and reduces overfitting. The final outcome is determined by averaging predictions in regression or selecting the most common class in classification. This approach improves reliability and handles noisy data better than a single decision tree. Random Forests work effectively with both numerical and categorical variables, though they can be computationally demanding and less interpretable than individual trees [43].

Gradient Boosting: An ensemble method for both classification and regression that builds a series of decision trees in sequence, with each new tree aiming to reduce the mistakes made by earlier ones. Instead of training trees independently (as in Random Forest), Gradient Boosting gradually improves performance by optimizing a loss function through gradient descent. This process allows the model to capture complex patterns and achieve high accuracy, though it also increases the risk of overfitting if not well-tuned. It performs well with both numerical and categorical data and requires little preprocessing, but it demands more computation and careful adjustment of parameters such as learning rate and tree depth to avoid overfitting [44].

XGBoost (Extreme Gradient Boosting): An improved version of Gradient Boosting that adds more efficiency and control. It uses the same idea of building trees step by step, but includes extra features like regularization to reduce overfitting, faster computation via parallel processing, and automatic handling of missing data. These improvements make XGBoost more stable, faster, and often more accurate than standard Gradient Boosting [45].

LightGBM (Light Gradient Boosting Machine): A fast and efficient gradient boosting method for classification and regression, similar to XGBoost but optimized for large datasets. It grows trees leaf-wise, focusing on the most significant splits to improve accuracy while using less memory. It automatically handles missing values and categorical features, making it well-suited for large-scale datasets, though it requires careful tuning to prevent overfitting [46].

CatBoost (Categorical Boosting): A gradient boosting algorithm designed to handle categorical features efficiently without manual encoding. Instead of growing trees independently (like Random Forest), or leaf-wise (like LightGBM), or level-wise (like XGBoost), CatBoost builds symmetrical trees where all leaves at the same depth are split using the same condition. This ensures balanced and regularized learning. It also uses ordered boosting to reduce overfitting and prevent data leakage. These features make CatBoost particularly effective for datasets with many categorical variables [47].

We also included logistic regression as a baseline model. While limited in capturing complex and non-linear relationships, its simplicity and clear interpretation make it a reliable benchmark, particularly in public health studies where it remains a standard approach.

### 3.8. Hyperparameter Tuning

To enhance the model’s performance, we fine-tuned the hyperparameters using Bayesian Optimization. Unlike Grid Search, which tests every possible combination of hyperparameters, Bayesian Optimization takes a ‘smarter approach’. It begins by sampling a small set of random hyperparameter values, then builds a probabilistic model to predict how changes in these values might impact performance. As it learns from previous results, it gradually narrows its search to focus on the combinations that are most likely to improve performance. This method is especially valuable for large search spaces, as it significantly reduces the number of iterations required to find the best-performing combination of hyperparameters [48].

To ensure robust tuning, we implemented 5-fold cross-validation. The training data was divided into five equal parts, and in each iteration, four parts were used for training while the fifth served as the validation set. By rotating the validation set across all five folds, we obtained performance metrics for every subset of the data. The final score was the average across all folds, providing a more reliable estimate of the model’s generalization ability.

The best hyperparameters were chosen based on the highest average F1 score for women who did not utilize SBA during cross-validation. This metric was selected to prioritize the accurate identification of this underrepresented and at-risk group. Salmi et al. [49] show that F1-score is widely used in medical diagnosis with imbalanced data because it balances precision and recall and accounts for misclassification of both majority and minority classes. Kumar et al. [50] emphasize that accuracy alone is misleading under imbalance, and that performance must be judged through precision and recall trade-offs. Ghanem et al. [51] highlight that the F1-score is especially useful in imbalanced settings since it captures the balance between precision and recall in a single measure. Finally, we tested the optimized model on a held-out test set to verify its performance on unseen data. This step was essential to ensure that the model was not overfitting to the training set and could generalize efficiently.

### 3.9. Model Evaluation

After hyperparameter tuning, each model was retrained on the entire training set (with the selected parameters) and evaluated on the held-out test set (20%). This final evaluation provides an unbiased estimate of real-world performance.

The following metrics were computed to assess model quality:

1. Accuracy: the proportion of correct predictions made by the model out of all predictions.

$$Accuracy=\frac{TP+TN}{TP+TN+FP+FN}$$

2. Area Under the ROC Curve (AUC): measures how well the model can distinguish between different classes; i.e., it measures how well the model separates users from non-users of SBA. It is a score ranging from 0 to 1, where 1 means perfect distinction and 0.5 means no distinction.
3. Recall: measures how well the model identifies positive cases. In this case, it gives the proportion of SBA cases correctly identified by the model.

$$Recall=\frac{TP}{TP+FN}$$

4. Precision: the proportion of true positive results out of all the positive results predicted by the model. It measures how many of the women predicted by the model as using SBA were correctly classified. In other words, it is the proportion of true SBA cases out of all cases the model predicted as SBA.

$$Precision=\frac{TP}{TP+FP}$$

5. F1-Score: combines precision and recall into a single score. It is useful for evaluating models trained on imbalanced data.

$$F1\;Score\;=\;2\times \frac{Precision\;\times \;Recall}{Precision\;+\;Recall}$$

where TP = True Positive, TN = True Negative, FP = False Positive, FN = False Negative.

### 3.10. Enhancing Model Interpretability with SHAP

SHAP (SHapley Additive Explanations) is a machine learning interpretability method that explains how each feature contributes to a model’s predictions. It assigns SHAP values to individual features, indicating their positive or negative impact on the predicted outcome. This allows us to understand not just which features are important, but how they influence the model’s decision. Unlike traditional feature importance methods, SHAP provides both global insights (overall feature importance) and local explanations (individual predictions). This makes it particularly useful in critical areas like healthcare, where model transparency is essential. By using SHAP, complex models such as Random Forest and XGBoost become more interpretable, helping stakeholders gain trust in the predictions and make informed decisions [52].

We used a combination of Stata 15, R 4.4.0, and Python 3.13 for the analysis. Initial data cleaning, outlier removal, and categorization of variables were conducted in Stata. Missing data imputation was performed in R using the VIM package. Tree-based machine learning modeling and interpretability analysis were carried out in Python. The scikit-learn library was used for decision tree, random forest, and gradient boosting models, while xgboost, lightgbm, and catboost were used for their respective algorithms. Bayesian optimization for hyperparameter tuning was implemented using the scikit-optimize library. For model interpretation, the shap library was used to generate both global and local explanations of feature contributions.

**Figure 3:**
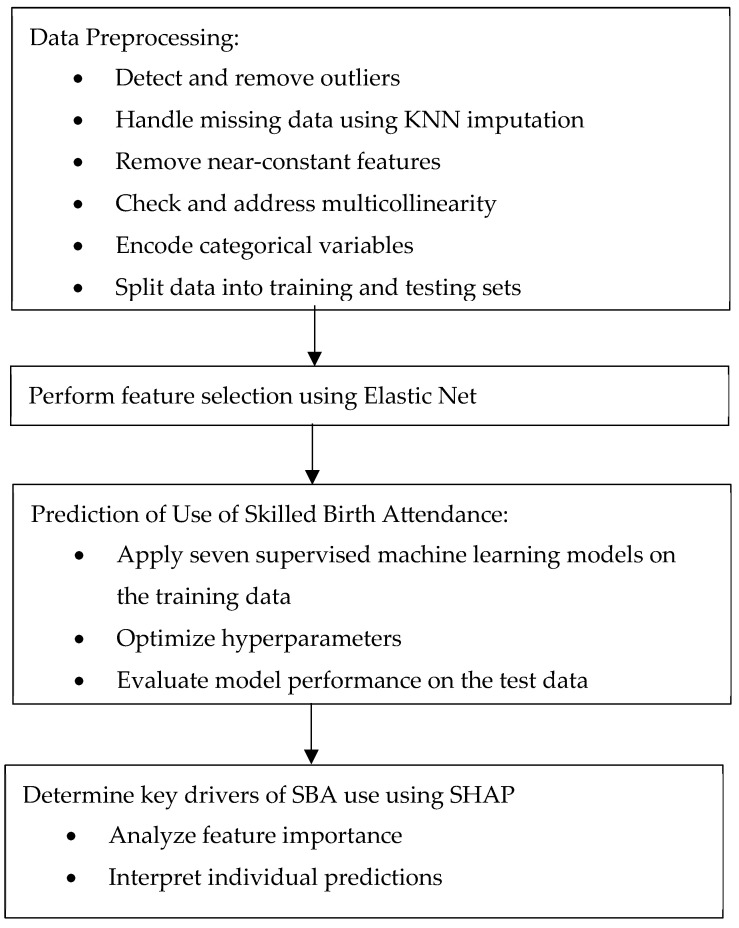
Workflow adopted in the study.

Figure 3 summarizes the workflow adopted in this study.

## 4. Results

### 4.1. Data Characteristics and Preprocessing Results

We analyzed 9611 women aged 15–49 who had given birth in the five years before the 2016 UDHS. The data was first examined for missingness. Out of the 40 features used in analysis, 9 features had missing data ranging from 0.03% to 2.8%. Missing data were imputed using KNN imputation. In the case of the health insurance variable, 98.93% of the records were “no” while only 1.07% were “yes.” It was, therefore, dropped from further analysis due to its being a near-zero variance variable.

With regard to multicollinearity, some high correlations were expected because of how the survey questions were structured. For instance, women not in a union were consistently grouped into single categories for variables such as partner’s education, partner’s employment, family type, and healthcare autonomy, since these questions were not applicable to them. Similarly, number of ANC visits and ANC in the first trimester overlapped because all women with zero ANC visits were automatically coded as not having a first-trimester visit.

**Figure 4:**
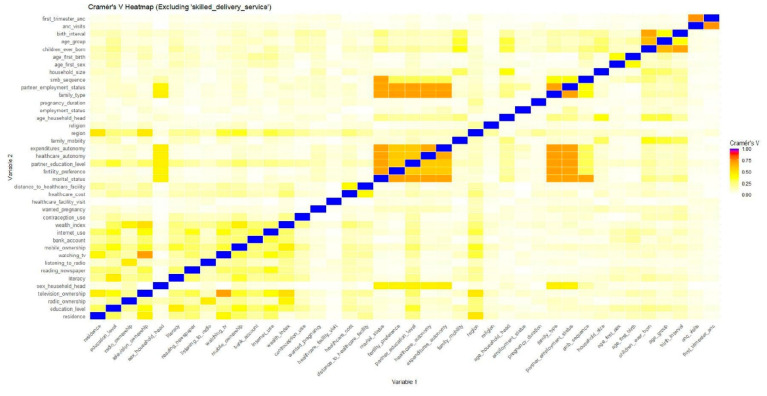
Feature Correlation Heatmap (Cramér’s V).

Despite these inherent correlations, the heatmap of pairwise Cramér’s V values indicated no perfect associations (Figure 4), and all variables were retained due to their theoretical importance in explaining maternal health-seeking behaviour. Dropping such variables would have risked losing conceptually relevant information, even if some correlations existed. In addition, the methodology we applied reduces concerns about multicollinearity. Elastic Net feature selection, by combining L1 and L2 regularization, shrinks redundant predictors and retains those with the greatest predictive value, thereby limiting the influence of highly correlated variables. Moreover, tree-based models such as Random Forest, Gradient Boosting, and CatBoost, which we employed, are inherently robust to multicollinearity [53]. Taken together, these approaches ensured that multicollinearity was not a major issue, and all theoretically important features were retained for modeling.

**Figure 5:**
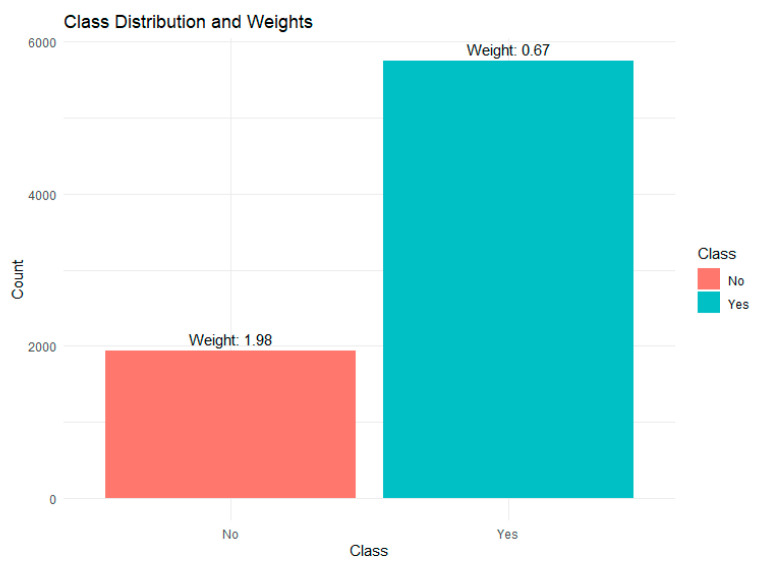
Class distribution and weight assignment.

Upon examining the distribution of the target variable in the training set, we identified an imbalance between the two classes in the target variable (SBA Yes/SBA No) (Figure 5). Class imbalance was handled using class weights in the study.

### 4.2. Socio-Demographic and Economic Characteristics of the Study Population

A total of 9611 women aged 15–49 who had given birth in the 5-year period preceding the 2016 UDHS were included in this study. According to results in Table 1, women in urban areas had a higher proportion of SBA use (90.69%) compared to those in rural areas (70.82%). Among rural women, 29.18% gave birth without skilled assistance, while in urban areas, only 9.31% lacked SBA. Women aged 15–19 years had the highest SBA utilization rate at 80.56%, while those aged 40 and older had the lowest SBA rate at 62.93%. Women in the richest quintile had the highest SBA rate at 87.30%, while those in the poorest quintile had the lowest at 68.14%. Almost a third (31.86%) of women in the poorest quintile gave birth without skilled care, compared to only 12.70% in the richest group.

### 4.3. Maternal Obstetric Characteristics of the Study Population

As seen in Table 2, women who gave birth for the first time had the highest SBA rate (86.73%) compared to those with two or more children. Women who had four or more ANC visits had the highest SBA rate at 80.70%, while those with fewer than four ANC visits had a lower SBA rate of 67.40.%. Over a half (54.92%) of women with no ANC visits gave birth without skilled assistance. Women with a birth interval of more than three years had the highest skilled birth attendance (SBA) rate at 77.04%, followed by those with a birth interval of less than two years at 70.43%. Women with a birth interval of 2–3 years had the lowest SBA rate at 67.55%, with 32.45% giving birth without skilled assistance.

### 4.4. Feature Selection

**Figure 6:**
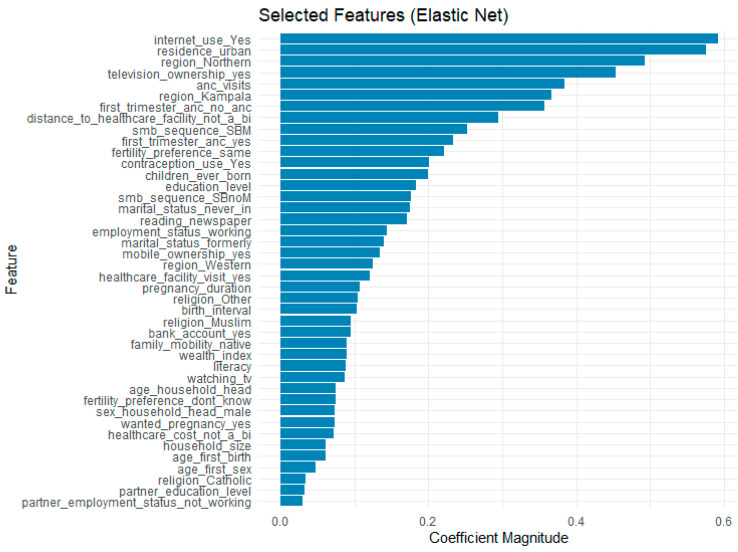
Features Selected by Elastic Net.

The feature selection process using Elastic Net regularization identified the most influential predictors of SBA usage while mitigating the effects of multicollinearity. By balancing L1 (Lasso) and L2 (Ridge) penalties, the model retained only the most relevant features, eliminating redundant or weak predictive variables. The selected features and their corresponding coefficient magnitudes are presented in Figure 6. Among the strongest predictors were internet use, urban residence, television ownership, number of ANC visits, and regional factors (e.g., residing in Northern Uganda or Kampala).

### 4.5. Prediction of Use of Skilled Birth Attendance

We applied seven supervised machine learning models, including Logistic Regression, Decision Tree, Random Forest, Gradient Boosting, XGBoost, LightGBM, and CatBoost, to predict skilled birth attendance (SBA). The dataset was split into training and test sets (80/20). Hyperparameters were optimized using Bayesian optimization with 5-fold cross-validation. To address class imbalance, we applied class weighting, giving more importance to women without SBA. Model performance was evaluated on the test set using accuracy, precision, recall, F1-score, and AUC, with the F1-score chosen as the key metric because it balances precision and recall.

In evaluating the performance of various models, we found that several performed quite similarly, especially in terms of recall, F1-score, and AUC. Our primary focus was on identifying women who did not use skilled birth attendance (SBA), which is the group of particular concern from a public health perspective. For this reason, we optimized all models using the F1-score for class 0 (women without SBA), which balances both precision and recall for this underrepresented group.

Across all models, XGBoost achieved the highest AUC (0.7473) and one of the best F1-scores (0.52), alongside Gradient Boosting, CatBoost, and LightGBM (Table 3). While the differences in performance were small, XGBoost stood out as the most consistent across all metrics including precision, recall, accuracy, and AUC, making it the most reliable choice overall.

The relatively modest F1-scores are largely due to the imbalance in the dataset, where fewer women fell into the “no SBA” category. To address this, we used class weighting, which gave more importance to these minority cases during training. This helped the models focus more on correctly identifying those most at risk of not accessing skilled care.

**Figure 7:**
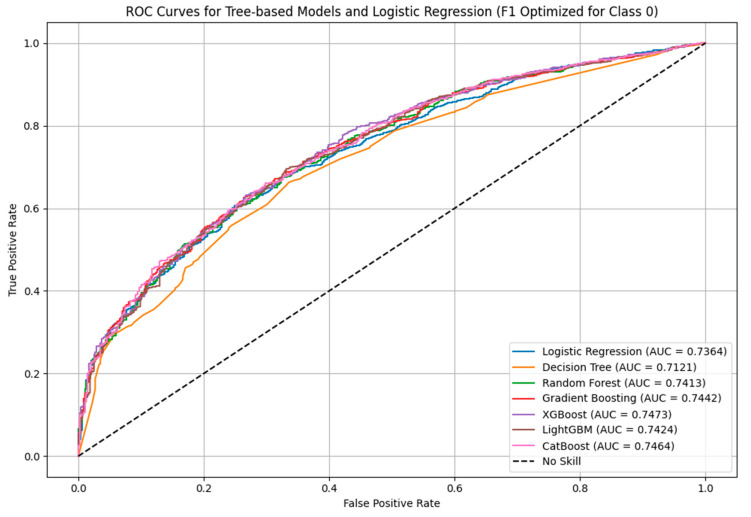
ROC Curve Comparison for Models.

Although Gradient Boosting, CatBoost, and LightGBM also performed well (Figure 7) and remain strong alternatives, XGBoost offered the best combination of performance and consistency for our objectives.

### 4.6. Key Drivers of SBA Use: SHAP Explanations

To better understand why the models made their predictions as they did, we applied SHAP (SHapley Additive exPlanations), an explainability method that assigns each feature a value showing its contribution to an individual prediction. Unlike other feature importance ranking scores, such as those in the Random Forest algorithm, SHAP allows us to see both the direction and the strength of each factor’s influence, making results transparent and easier to interpret. By using SHAP, we can identify which socio-demographic and obstetric variables most strongly shaped the likelihood of skilled birth attendance (SBA) in Uganda.

**Figure 8:**
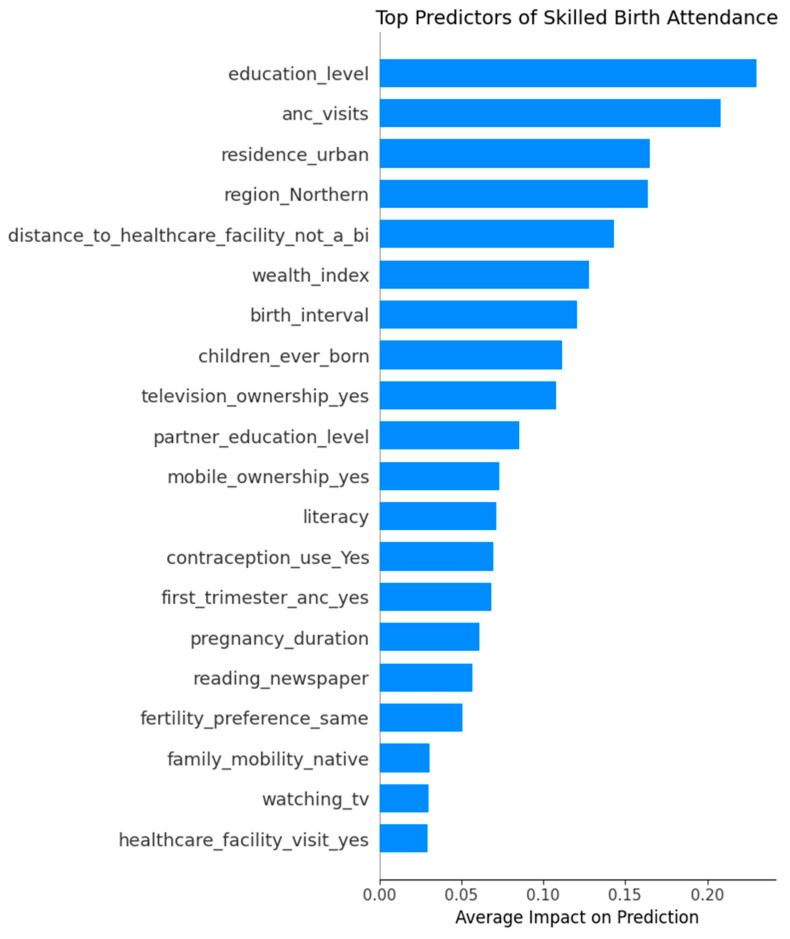
Key Predictors of Skilled Birth Attendance: SHAP-Based Feature Importance.

The SHAP feature importance plot (Figure 8) highlights the most influential variables driving the model’s predictions for skilled birth attendance. Education level emerged as the most critical factor, followed closely by number of ANC visits and urban residence, emphasizing the role of education and healthcare access in maternal care decisions. Regional disparities, particularly for the non-Northern regions, and perceived distance to healthcare facilities also significantly impacted predictions. The Sex–Marriage–Birth (SMB) sequence variable, which captures the order of key life events, did not rank among the top 20 predictors of SBA use. These insights not only enhance model transparency but also point to key intervention areas for improving maternal health outcomes.

**Figure 9:**
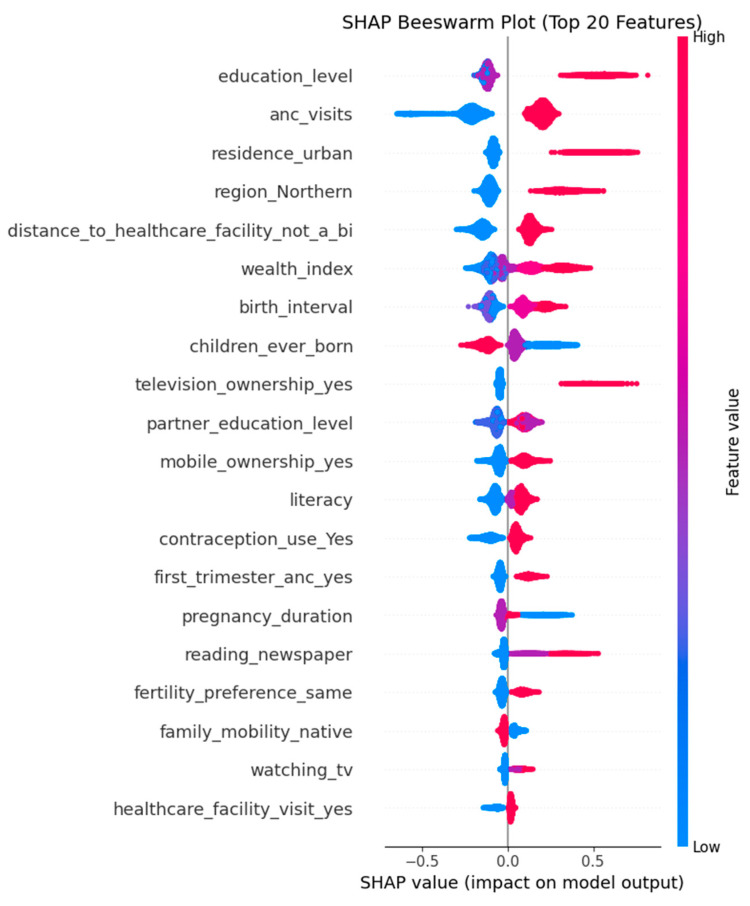
SHAP Beeswarm Plot Showing the Top 20 Predictors of Skilled Birth Attendance.

The SHAP beeswarm plot (Figure 9) summarizes the 20 most important features influencing the model’s predictions of SBA use. Features are ranked from top to bottom by their overall importance. Each dot represents one woman in the dataset. The colour indicates the feature magnitude (blue = low, red = high), while the position on the x-axis shows the SHAP value. A SHAP value measures how much a feature pushes the prediction toward SBA use (positive, right side) or away from SBA use (negative, left side). In simple terms, a SHAP value shows how much each factor increases or decreases the model’s prediction for an individual woman, making the results easier to interpret. For example, higher education level and more ANC visits shifted predictions toward SBA use, while lower values of these features shifted predictions toward non-use. This visualization, therefore, shows both the strength and the direction of influence for each predictor.

The top ten insights are:

1. Education level is the most important factor. Women with higher education are much more likely to use skilled delivery services.
2. ANC visits also play a big role. Women who attended more antenatal care visits, especially 4 or more, have higher chances of using skilled birth attendants.
3. Urban residence is linked to higher use of services.
4. Region (Northern) has positive SHAP values, meaning women from the Northern region are more likely to use skilled delivery services, according to the model.
5. Distance to health facilities is another important factor. Women who said distance was a big problem were less likely to use skilled care.
6. Wealth index shows that wealthier women are more likely to use skilled services.
7. Longer birth intervals are linked to higher chances of use of SBA.
8. Number of children ever born shows that first-time mothers or those with fewer children are more likely to seek skilled delivery services.
9. Television ownership and other media exposure like mobile ownership help improve use of services.
10. Partner’s education also matters; women with more educated partners were more likely to use skilled care.

**Figure 10:**
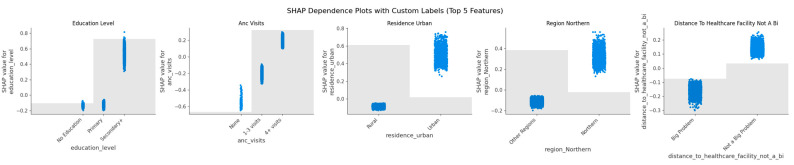
SHAP Dependence Plots Showing How the Top 5 Predictors Influence Skilled Birth Attendance.

The SHAP dependence plots (Figure 10) further simplify the visualizations for the top five features. Each point in the graphs represents one woman in the dataset, and the SHAP value on the y-axis shows how much that feature influenced the model’s prediction toward SBA use (positive values) or away from SBA use (negative values). The graphs have been interpreted below:

1. Education level: Women with secondary or higher education strongly increase the likelihood of SBA use compared to those with no or only primary education.
2. ANC visits: More antenatal visits, especially 4 or more, increase SBA use, while no visits reduce it.
3. Residence (urban): Living in urban areas shifts predictions toward SBA, while rural areas shift predictions away.
4. Region (Northern): Being from the Northern region increases the likelihood of SBA use compared to other regions.
5. Distance to healthcare facility: Women who perceive distance as a big problem are less likely to use SBA, while those who report no distance problem are more likely to access skilled delivery.

Together, these plots confirm that education, ANC attendance, place of residence, region, and distance barriers are the most influential drivers of SBA use in Uganda.

## 5. Discussion

This study set out to develop interpretable machine learning models to predict the likelihood of skilled birth attendance (SBA) using nationally representative demographic and health survey data from Uganda. We evaluated a range of models, including logistic regression and six tree-based classifiers: decision tree, random forest, gradient boosting, XGBoost, LightGBM, and CatBoost. Among these, the best-performing model, XGBoost, achieved an F1-score of 0.52, a recall of 0.73, and an AUC of 0.75. While these figures may not appear exceptionally high, they reflect the intrinsic challenge of predicting imbalanced health outcomes [29,54,55,56,57].

Whereas our F1 is 0.52, our AUC is 0.75, which shows good separation between users and non-users of SBA and is in line with other country-specific maternal healthcare-seeking behavior studies. For example, a study in Zanzibar also reported moderate results (AUC of 0.74–0.80) when predicting place of delivery in the presence of imbalanced data [29]. Similarly, a large multi-country study on the maternal and child health continuum also showed a severely imbalanced target (≈90% NO) and found low specificity (51.6%) and moderate AUC (≈0.70) despite a much larger dataset [57]. In addition, a recent 12-country East African analysis predicting home delivery after ANC visits reported Random Forest AUC values of only 0.68–0.69, even after resampling to balance the classes. These consistent findings highlight that moderate performance evaluation metrics are expected when addressing maternal health outcomes under imbalance.

On the other hand, our results may be lower than those of other studies, mainly due to differences in study design. For example, Ngusie et al. and Taye et al. [32,33,56] used data from 12 and 27 sub-Saharan African countries, respectively, to build machine learning models for the prediction of place of delivery and SBA use. While this wide scope helps with generalizing results across countries, it does not allow for deeper insights within any single country. Their studies also reported the highest AUC (AUC > 90%), which may partly be due to the larger, combined datasets, which boosted performance. In contrast, our study focuses only on Uganda, which means we can capture country-specific patterns that might otherwise be missed. For instance, we found that being from the Northern region was one of the strongest predictors of skilled birth attendance. This kind of detail would likely have been lost in a combined dataset. Focusing on one country helps ensure that the findings are relevant to national health policies and targeted interventions. It is also worth noting that the study done by Ngusie et al. [32] used place of delivery as their target variable. In contrast, we focused on the use of SBA as the outcome, a choice that aligns with WHO recommendations for assessing maternal healthcare [58]. Our data show that skilled attendance can occur outside of health facilities, for example, through home visits by trained providers.

It is also important to highlight key methodological differences between our work and previous studies. For instance, Tesfaye et al. and Taye et al. [30,33] included “place of delivery” as a predictor for skilled delivery service use, which is an approach that risks data leakage, since place of delivery is inherently tied to the outcome being predicted. We deliberately excluded such variables to ensure that our model’s predictions were based only on true predictors known before delivery.

Our study achieved results that are slightly lower than those reported by Fredriksson et al. [29] (AUC of 0.74–0.80). One possible reason for this difference is the availability of certain variables, such as place of previous delivery, which was used in their model. In our dataset, this variable was missing for more than half of the observations and was therefore excluded from our analysis. Importantly, while their study focused solely on prediction, we took a step further by applying SHAP (SHapley Additive exPlanations) to interpret both individual-level and overall model predictions. This helped us understand not only what the model predicted, but also why. This is an essential step in making machine learning outputs useful for real-world decision-making.

Additionally, to address class imbalance, we applied class weighting during model training. While techniques like Synthetic Minority Oversampling Technique (SMOTE) are commonly used in similar research, they are designed for continuous variables and can produce unrealistic synthetic examples when applied to categorical data [39]. Given that our dataset was entirely categorical, class weighting was a more appropriate and reliable approach. By deliberately excluding predictors tied directly to the outcome, such as place of delivery, and by applying class weighting to handle imbalance, we ensured that the models were trained on meaningful and unbiased predictors. This strengthens the credibility of our findings and enhances their practical relevance for informing maternal health policy.

SHAP analysis identified five main factors that most strongly influenced whether a woman used skilled birth attendance (SBA): her level of education, the number of antenatal care (ANC) visits she attended, her region of residence (particularly Northern Uganda), whether she perceived distance to a health facility as a barrier, and whether she lived in an urban or rural area. Women with secondary education were more likely to use SBA, reflecting the well-established role of education in promoting health awareness, autonomy, and service utilization [59]. Similarly, women who attended four or more ANC visits were much more likely to use SBA, likely due to increased contact with health professionals and exposure to awareness campaigns [19].

Regional variation also played a key role in the decision to utilize SBA; women from Northern Uganda were more likely to access SBA services. Even though our descriptive analysis showed that Northern Uganda had relatively lower SBA coverage compared to Central regions, both SHAP values and partial dependence plots indicated that, when adjusting for other socio-demographic and obstetric factors, women from Northern Uganda had a higher likelihood of SBA use compared to women from other regions. This finding aligns with earlier work by Sserwanja et al. [8], who reported that women in the northern region were three times more likely than those in the Central region to utilize health facilities during childbirth, despite Kampala having a higher concentration of health facilities and healthcare workers. This could be in part due to the region receiving substantial humanitarian interventions targeting maternal health following civil conflict, including free services in internally displaced persons’ camps, where many people were residing [60]. Our findings, therefore, suggest that while absolute use remains lower in the north, once other barriers are accounted for, women in this region may be more likely to use SBA services compared to their counterparts elsewhere.

Further, women who reported that distance was not a barrier had higher SBA use, underlining the importance of physical access to services [20]. Finally, urban residence was associated with greater SBA use, emphasizing the disparity between urban and rural areas in accessing skilled delivery services, probably due to better facility coverage, higher awareness, and improved transportation options in urban settings [20]. The SMB sequence variable was not a strong predictor in our model, but it is still conceptually important. The order of life events (such as sex before marriage or childbirth before marriage) reflects social norms that can shape maternal healthcare decisions. While it did not play a major role in prediction, it points to pathways that may be useful for designing culturally sensitive interventions.

Our findings have practical policy implications. For example, the strong influence of education suggests that expanding access to secondary schooling for girls could have significant effects on SBA uptake. Similarly, the importance of ANC visits highlights the need for community outreach programs that encourage early and regular ANC attendance, especially in rural settings where physical access is a barrier. Regional differences, particularly the higher likelihood of SBA use in Northern Uganda, point to the value of sustaining and scaling successful interventions such as free services in hard-to-reach areas. Addressing access barriers is equally important; outreach maternity services such as mobile clinics or health teams and transport support schemes such as motorcycle ambulances are urgently required in areas with remote communities to improve access to skilled birth attendants. Together, these examples illustrate how model outputs can guide targeted interventions and more efficient allocation of resources to groups most at risk of not using SBA.

It is important to note that, given the XGBoost model achieved an F1-score of 0.52, false negatives remain a concern. In this context, a false negative means that a woman who did not use SBA was misclassified as a user, potentially leading to missed opportunities for targeted interventions. This limitation is important because, although more women in the dataset reported SBA use, the smaller group of non-users represents the highest-risk population for negative maternal and neonatal outcomes. However, the relatively strong AUC (0.75) demonstrates that the model was able to distinguish users from non-users with good separation. More importantly, the primary contribution of this study was not prediction accuracy alone but the use of explainability methods such as SHAP to identify the key socio-demographic and obstetric drivers of SBA use. These insights remain valuable for informing maternal health policy, even if predictive performance is moderate.

## 6. Conclusions

This study applied interpretable machine learning to predict skilled birth attendance (SBA) in Uganda, using nationally representative DHS data. By applying SHAP, we were able to explain not just the model predictions but also the factors driving them, making the findings transparent and useful for policy and practice. Among the models tested, XGBoost performed best, achieving an F1-score of 0.52 and an AUC of 0.75, results consistent with other studies addressing imbalanced health data.

Our analysis showed that SBA use in Uganda is strongly influenced by education, antenatal care visits, region, urban–rural residence, and perceived distance to health facilities. Women with higher education and more ANC visits were more likely to use skilled attendants, while those in rural areas and those reporting distance as a barrier were less likely. Regional differences also emerged, with women in Northern Uganda showing higher likelihood of SBA use once other factors were considered.

These findings suggest that policies should go beyond expanding physical access and also address education, awareness, and regional disparities. Interventions should be tailored to rural and less educated women through strategies such as strengthened ANC outreach, transport support schemes, and community-based programs. By focusing on these groups, Uganda can make meaningful progress toward increasing skilled attendance at birth and improving maternal outcomes.

## Figures and Tables

**Table 1:** Socio-Demographic and Economic Characteristics of the Women in the Study by Utilization of SBA.

Variable	Category	SBA Yes (74.73%)	SBA No (25.27%)	Total
Family Mobility	Native	4201 (71.36%)	1686 (28.64%)	5887
	Internal Immigrant	2981 (80.05%)	743 (19.95%)	3724
Residence	Rural	5467 (70.82%)	2253 (29.18%)	7720
	Urban	1715 (90.69%)	176 (9.31%)	1891
Region	Central	1216 (78.91%)	325 (21.09%)	1541
	Kampala	467 (96.09%)	19 (3.91%)	486
	Northern	1876 (76.95%)	562 (23.05%)	2438
	Western	1760 (70.26%)	745 (29.74%)	2505
	Eastern	1863 (70.54%)	778 (29.46%)	2641
Religion	Anglican	2214 (73.87%)	783 (26.13%)	2997
	Catholic	2929 (74.26%)	1015 (25.74%)	3944
	Muslim	966 (81.24%)	223 (18.76%)	1189
	Other	1073 (72.45%)	408 (27.55%)	1481
Literacy	None	2542 (66.32%)	1291 (33.68%)	3833
	Partial	886 (71.74%)	349 (28.26%)	1235
	Complete	3754 (82.63%)	789 (17.37%)	4543
Education Level	No Education	789 (64.99%)	425 (35.01%)	1214
	Primary	4147 (70.24%)	1757 (29.76%)	5904
	Secondary	2246 (90.09%)	247 (9.91%)	2493
Wealth Index	Poorest	1608 (68.14%)	752 (31.86%)	2360
	Poorer	1410 (69.7%)	613 (30.3%)	2023
	Middle	1372 (73.37%)	498 (26.63%)	1870
	Richer	1424 (79.51%)	367 (20.49%)	1791
	Richest	1368 (87.30%)	199 (12.7%)	1567
Age Group	15–19	1235 (80.56%)	298 (19.44%)	1533
	20–24	2136 (77.7%)	613 (22.3%)	2749
	25–29	1764 (75.51%)	572 (24.49%)	2336
	30–34	1088 (70.93%)	446 (29.07%)	1534
	35–39	684 (66.93%)	338 (33.07%)	1022
	40++	275 (62.93%)	162 (37.07%)	437
Employment Status	Not Working	1206 (78.62%)	328 (21.38%)	1534
	Working	5976 (73.99%)	2101 (26.01%)	8077
Marital Status	Currently in Union	5910 (74.16%)	2059 (25.84%)	7969
	Formerly in Union	819 (72.93%)	304 (27.07%)	1123
	Never In Union	453 (87.28%)	66 (12.72%)	519
SMB Sequence	MSB	2987 (72.59%)	1128 (27.41%)	4115
	SBM	1191 (78.30%)	330 (21.70%)	1521
	SMB	2551 (73.81%)	905 (26.19%)	3456
	SBnoM	453 (87.28%)	66 (12.72%)	519
Family Type	Monogamous	4468 (74.77%)	1508 (25.23%)	5976
	Polygamous	1442 (72.35%)	551 (27.65%)	1993
	Not In Union	1272 (77.47%)	370 (22.53%)	1642
Household Size	1–4	2555 (78.62%)	695 (21.38%)	3250
	5–8	3554 (72.63%)	1339 (27.37%)	4893
	>8	1073 (73.09%)	395 (26.91%)	1468
Sex Household Head	Female	1906 (76.15%)	597 (23.85%)	2503
	Male	5276 (74.23%)	1832 (25.77%)	7108
Age of Household Head	<25	738 (75.93%)	234 (24.07%)	972
	25–29	1333 (77.05%)	397 (22.95%)	1730
	30–49	3990 (73.06%)	1471 (26.94%)	5461
	50++	1121 (77.42%)	327 (22.58%)	1448
Radio Ownership	No	2977 (70.51%)	1245 (29.49%)	4222
	Yes	4205 (78.03%)	1184 (21.97%)	5389
Television Ownership	No	5933 (71.65%)	2347 (28.35%)	8280
	Yes	1249 (93.84%)	82 (6.16%)	1331
Mobile Ownership	No	3895 (68.84%)	1763 (31.16%)	5658
	Yes	3287 (83.15%)	666 (16.85%)	3953
Bank Account Ownership	No	6262 (73.27%)	2285 (26.73%)	8547
	Yes	920 (86.47%)	144 (13.53%)	1064
Internet Use	No	6708 (73.52%)	2416 (26.48%)	9124
	Yes	474 (97.33%)	13 (2.67%)	487
Reading Newspaper	At Least Once a Week	536 (92.89%)	41 (7.11%)	577
	Less Than Once a Week	852 (86.23%)	136 (13.77%)	988
	Not At All	5794 (72.01%)	2252 (27.99%)	8046
Listening To Radio	At Least Once a Week	4213 (77.4%)	1230 (22.6%)	5443
	Less Than Once a Week	1170 (77.02%)	349 (22.98%)	1519
	Not At All	1799 (67.91%)	850 (32.09%)	2649
Watching Tv	At Least Once a Week	1303 (91.25%)	125 (8.75%)	1428
	Less Than Once a Week	741 (79.25%)	194 (20.75%)	935
	Not At All	5138 (70.89%)	2110 (29.11%)	7248
Health Insurance	No	7089 (74.56%)	2419 (25.44%)	9508
	Yes	93 (90.29%)	10 (9.71%)	103
Partner Education Level	No Education	436 (68.55%)	200 (31.45%)	636
	Primary	3001 (67.94%)	1416 (32.06%)	4417
	Secondary	2473 (84.81%)	443 (15.19%)	2916
	Not In Union	1272 (77.47%)	370 (22.53%)	1642
Partner Employment Status	Not Working	194 (68.55%)	89 (31.45%)	283
	Working	5716 (74.37%)	1970 (25.63%)	7686
	Not In Union	1272 (77.47%)	370 (22.53%)	1642
Healthcare Facility Visit in the Past Year	No	1415 (71.11%)	575 (28.89%)	1990
	Yes	5767 (75.67%)	1854 (24.33%)	7621
Healthcare Cost	Big Problem	3337 (70.64%)	1387 (29.36%)	4724
	Not A Big Problem	3845 (78.68%)	1042 (21.32%)	4887
Distance To Healthcare Facility	Big Problem	2704 (67.67%)	1292 (32.33%)	3996
	Not A Big Problem	4478 (79.75%)	1137 (20.25%)	5615
Healthcare Decision-Making	Husband/Partner Alone	1581 (73.36%)	574 (26.64%)	2155
	Respondent Alone	1697 (73.78%)	603 (26.22%)	2300
	Respondent And Husband/Partner	2632 (74.9%)	882 (25.1%)	3514
	Not In Union	1272 (77.47%)	370 (22.53%)	1642
Expenditures Decision-Making	Husband/Partner Alone	2086 (73.17%)	765 (26.83%)	2851
	Respondent Alone	883 (72.32%)	338 (27.68%)	1221
	Respondent And Husband/Partner	2941 (75.47%)	956 (24.53%)	3897
	Not In Union	1272 (77.47%)	370 (22.53%)	1642

**Table 2:** Maternal Obstetric Characteristics of the Women in the Study by Utilization of SBA.

Variable	Category	SBA Yes (74.73%)	SBA No (25.27%)	Total
Contraception Use	No	1968 (68.76%)	894 (31.24%)	2862
	Yes	5214 (77.26%)	1535 (22.74%)	6749
Wanted Pregnancy	No	3118 (72.56%)	1179 (27.44%)	4297
	Yes	4064 (76.48%)	1250 (23.52%)	5314
Wanting Same Number of Children as Husband/Partner	No	2542 (72.94%)	943 (27.06%)	3485
	Yes	2267 (78.01%)	639 (21.99%)	2906
	Don’t Know	1101 (69.77%)	477 (30.23%)	1578
	Not In Union	1272 (77.47%)	370 (22.53%)	1642
Children Ever Born	1	1595 (86.73%)	244 (13.27%)	1839
	2–4	3332 (76.76%)	1009 (23.24%)	4341
	5++	2255 (65.72%)	1176 (34.28%)	3431
Birth Interval (Years)	<2	1203 (70.43%)	505 (29.57%)	1708
	2–3	2048 (67.55%)	984 (32.45%)	3032
	>3	2335 (77.04%)	696 (22.96%)	3031
	First Birth	1596 (86.74%)	244 (13.26%)	1840
Age at First Sex	Early	1211 (67.84%)	574 (32.16%)	1785
	Moderate	3579 (74.04%)	1255 (25.96%)	4834
	Late	2392 (79.95%)	600 (20.05%)	2992
Age at First Birth	<18	2611 (70.85%)	1074 (29.15%)	3685
	18–24	4202 (76.65%)	1280 (23.35%)	5482
	>25	369 (83.11%)	75 (16.89%)	444
Number of ANC Visits	None	119 (45.08%)	145 (54.92%)	264
	1–3	2431 (67.40%)	1176 (32.60%)	3607
	4++	4632 (80.70%)	1108 (19.30%)	5740
First Trimester ANC	No	4737 (73.07%)	1746 (26.93%)	6483
	Yes	2281 (81.23%)	527 (18.77%)	2808
	No ANC	164 (51.25%)	156 (48.75%)	320
Pregnancy Duration (Months)	<9	1117 (79.62%)	286 (20.38%)	1403
	9	5464 (73.82%)	1938 (26.18%)	7402
	>9	601 (74.57%)	205 (25.43%)	806
Place of Delivery	Health facility	6971 (98.24)	125 (1.76)	7096
	Not at health facility	211 (8.39)	2304 (91.61)	2515

**Table 3:** Comparison of the Performance of Machine Learning Models in the Prediction of SBA.

Method	Precision	Recall	F1-Score	Accuracy	AUC
Logistic Regression	0.39	0.76	0.51	0.64	0.7364
Random Forest	0.40	0.71	0.51	0.66	0.7413
Gradient Boosting	0.40	0.74	0.52	0.65	0.7442
XGBoost	0.40	0.73	0.52	0.66	0.7473
LightGBM	0.43	0.67	0.52	0.69	0.7424
Decision Tree	0.40	0.66	0.50	0.66	0.7121
CatBoost	0.41	0.70	0.52	0.67	0.7464

## Data Availability

This study used secondary data. The dataset utilized in this study is publicly available through the DHS website upon request to the relevant authority (https://dhsprogram.com/data/available-datasets.cfm, accessed on 20 September 2024). Furthermore, the code used in this study can be provided upon reasonable request to the corresponding author.
